# Mining disease genes using integrated protein–protein interaction and gene–gene co-regulation information

**DOI:** 10.1016/j.fob.2015.03.011

**Published:** 2015-03-27

**Authors:** Jin Li, Limei Wang, Maozu Guo, Ruijie Zhang, Qiguo Dai, Xiaoyan Liu, Chunyu Wang, Zhixia Teng, Ping Xuan, Mingming Zhang

**Affiliations:** a School of Computer Science and Technology, Harbin Institute of Technology, Harbin, Heilongjiang, China; b School of Life Science and Technology, Harbin Institute of Technology, Harbin, Heilongjiang, China; c College of Bioinformatics Science and Technology, Harbin Medical University, Harbin, Heilongjiang, China; d School of Basic Medical Sciences, Harbin Medical University, Harbin, Heilongjiang, China

**Keywords:** AD, Alzheimer disease, eQTLs, expression quantitative trait loci, GGCRN, gene–gene co-regulation network, HPRD, Human Protein Reference Database, KEGG, Kyoto Encyclopedia of Genes and Genomes, PPI, protein–protein interaction, RWR, random walk with restart, SNP, single-nucleotide polymorphism, eQTL, Co-regulation network, Disease gene mining, Protein–protein interaction, Random walk with restart

## Abstract

In humans, despite the rapid increase in disease-associated gene discovery, a large proportion of disease-associated genes are still unknown. Many network-based approaches have been used to prioritize disease genes. Many networks, such as the protein–protein interaction (PPI), KEGG, and gene co-expression networks, have been used. Expression quantitative trait loci (eQTLs) have been successfully applied for the determination of genes associated with several diseases. In this study, we constructed an eQTL-based gene–gene co-regulation network (GGCRN) and used it to mine for disease genes. We adopted the random walk with restart (RWR) algorithm to mine for genes associated with Alzheimer disease. Compared to the Human Protein Reference Database (HPRD) PPI network alone, the integrated HPRD PPI and GGCRN networks provided faster convergence and revealed new disease-related genes. Therefore, using the RWR algorithm for integrated PPI and GGCRN is an effective method for disease-associated gene mining.

## Highlights

• An eQTL-based gene–gene co-regulation network was constructed.
• We adopted a random walk with restart (RWR) algorithm to mine for Alzheimer-disease related genes.
• The integrated HPRD PPI and GGCRN network had faster convergence than using HPRD PPI alone.
• The integrated network also revealed new disease-related genes.

## 1 Introduction

In humans, despite the rapid increase in the discovery of disease–associated genes, the molecular basis of many diseases is still known. Even for diseases for which the molecular basis is partially understood, a large proportion of the associated genes are still unknown. The known disease-associated genes have been reported to represent only a very small proportion of the actual number of disease-associated genes [1,2]. Hence, mining for disease genes remains important.

Network-based approaches to human disease have multiple biological and clinical applications [3,4]. Many molecular networks have been constructed experimentally to characterize the physical and/or functional interactions between biomolecules [4,5]. There are many methods for disease gene mining using molecular networks, such as the direct neighborhood [6–13], Shortest path length [13–16], Diffusion kernel [8], random walk with restart [8,9,17], propagation flow [18], and clique backbone [19] methods. The random walk with restart (RWR) method has been reported to have the best performance in terms of precision and recall, while both the random walk and propagation flow methods are superior to the clustering and neighborhood methods [20,21]. The most useful network is the protein–protein interaction (PPI) network [6,8,9]. Some other resources are also used in disease gene mining, such as gene ontology, gene co-expression network, KEGG, structure, and TRANSFAC [7,10,15,16].

Expression quantitative trait loci (eQTLs) analyses of DNA use hundreds of thousands of single-nucleotide polymorphism (SNP) markers that capture human genetic variation [22]. This strategy has been successfully applied to several diseases, such as celiac disease [23], asthma [24] and type 2 diabetes [25]. An eQTL is a locus that regulates a gene expression phenotype [26]. If two genes are regulated by one or more of the same SNPs, they are considered to be co-regulated. Obviously, this co-regulation is only one type of gene interactions. We constructed a gene–gene co-regulation network (GGCRN) using eQTL data and believe that it will be useful for disease gene mining.

In this study, we developed a GGCRN, integrated it with the PPI network, and used the RWR method to mine for candidate disease genes. Using Alzheimer disease (AD) as an example, we demonstrated that this newly developed GGCRN is an effective resource for disease gene mining.

## 2 Materials and methods

### 2.1 Materials

#### 2.1.1 Protein–protein interaction data

The Human Protein Reference Database (HPRD) describes interaction networks in the human proteome [27]. All information in the HPRD has been manually extracted from the literature by expert biologists who read, interpreted and analyzed the published data. For this study, we used HPRD, release 9, which contains 38,989 protein–protein interactions among 9605 proteins.

#### 2.1.2 EQTL data

We used human brain tissue data for this disease gene mining study. The data were obtained from a series of 193 neuropathologically normal human brain samples using the Affymetrix GeneChip Human Mapping 500 K Array Set and Illumina HumanRefseq-8 Expression BeadChip platforms [28]. The eQTLs were determined by Matrix eQTL [29]. In this study, the cis-eQTL definition was a SNP within the gene body +1 Mb up/down stream of the gene body. We calculated cis-eQTLs and trans-eQTLs and performed FDR adjustment (*q* value < 0.1) separately; then we combined the cis-eQTLs and trans-eQTLs. Finally, we obtained 25,866 significant SNP-gene association pairs of 3709 genes. The results can be downloaded from the seeQTL database [30].

#### 2.1.3 AD-related genes

Online Mendelian Inheritance in Man (OMIM) is a comprehensive, authoritative compendium of human genes and genetic phenotypes that is freely available and updated daily. AD is classified as a neurodegenerative disorder, and it is associated with plaques and tangles in the brain [31]. We obtained 29 AD-related terms from OMIM [32]. After removing the terms with no approved gene symbol, we obtained 15 AD-related genes. Of these 15 genes, 14, 4 and 14 genes were present in the HPRD PPI, the GGCRN, and the HPRD PPI and GGCRN integrated network (Union network). We used the 14 genes that were present in the Union network for the subsequent analyses. See Supplemental Table 1.

### 2.2 Methods

#### 2.2.1 Gene–gene co-regulation network construction

The human brain data included 25,866 significant SNP-gene association pairs of 3709 genes. For each gene, we first extracted the SNPs that regulate it, and we called these significant related SNPs. If a SNP regulated two genes, we called it a common SNP of the two genes. We considered two genes to be co-regulated if a specific proportion of SNPs regulated both genes. Mathematically, for any 2 genes ($G_{i}$ and $G_{j}$), there are $n_{1}$ and $n_{2}$ significant related SNPs, respectively. The gene–gene co-regulation coefficient is defined as

$$\text{coreco}(G_{i}\text{,}G_{j})=\frac{\#(\text{SNPs}\;\text{in}\;G_{i}\cap \text{SNPs}\;\text{in}\;G_{j})}{\#(\text{SNPs}\;\text{in}\;G_{i}\cup \text{SNPs}\;\text{in}\;G_{j})}\text{,}$$

Where # (*A*) is the element number in set *A*. In other words, $\#(\text{SNPs}\;\text{in}\;G_{i}\cap \text{SNPs}\;\text{in}\;G_{j})$ is the number of common SNPs that regulate both gene $G_{i}$ and $G_{j}$; and $\#(\text{SNPs}\;\text{in}\;G_{i}\cup \text{SNPs}\;\text{in}\;G_{j})$ is the number of SNPs that regulate gene $G_{i}$ or $G_{j}$. For example, if $G_{i}$ and $G_{j}$ have 100 and 80 significant related SNPs, respectively, and 30 of them are common SNPs for $G_{i}$ and $G_{j}$, then the co-regulation coefficient is 30/(100 + 80 − 30) = 0.2. After calculating all co-regulation coefficients for all gene pairs, a reasonable threshold value for filtering the significant co-regulated gene pairs had to be established; for this purpose, we used the clustering coefficient difference maximization method [33]. The main function of this method is the determination of the difference in the maximum clustering coefficient difference between a real network and a random network if the real network is highly credible. Finally, we obtained the GGCRN using the significant co-regulated gene pairs.

#### 2.2.2 Random walk with restart algorithm

In this paper, we focused on the genetic data resources rather than on statistical methods. Therefore, we adopted a classic and efficient method. The random walk algorithm (RW) for graphs is defined as an iterative walker’s random transition from its current node to a neighboring node, and this is initiated at a given source node [34,35]. The random walk with restart algorithm (RWR) [36] is a variant of the random walk that allows for the restart of the walk at every time step at source node *s* with probability *r*. Formally, the RWR is defined as:

$$p^{t+1}=(1-r){\mathrm{Wp}}^{t}+{\mathrm{rp}}^{0}$$

where *W* is the column-normalized adjacency matrix of the graph and *p^t^* is a vector in which the *i*th element holds the probability of being at node *i* at time step *t*. A special case is the initial probability vector, *p*^0^, which is the probability of being at source node, *s*. In our application, *p*^0^ was constructed such that equal probabilities were assigned to the known disease genes, with the sum of the probabilities equal to 1. Genes were ranked according to the values in the steady-state probability vector *p^N^*. This was obtained by performing the iteration until the change between *p^t^* and *p^t^*^+1^ fell below 10^−6^. The results of RWR are affected by the restart probability, *r*. We perform a numerical experiment to select the proper *r* value.

**Fig. 1:**
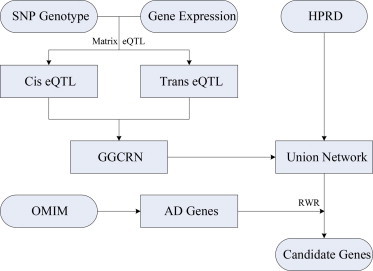
The flow chart of RWR method using the Union network.

The flow chart is illustrated in Fig. 1.

#### 2.2.3 Direct neighborhood algorithm

We also compared the RWR method with the direct neighborhood (DN) method. In the DN method, the interaction partners in the network were determined for each known disease gene. The more linkages that exist between a gene and known disease genes for a particular disease, the greater the possibility that it is related to that disease. In this study, genes with more than 1 linkage with known disease genes were considered candidate disease genes.

## 3 Results and discussion

### 3.1 Gene–gene co-regulation network

**Fig. 2:**
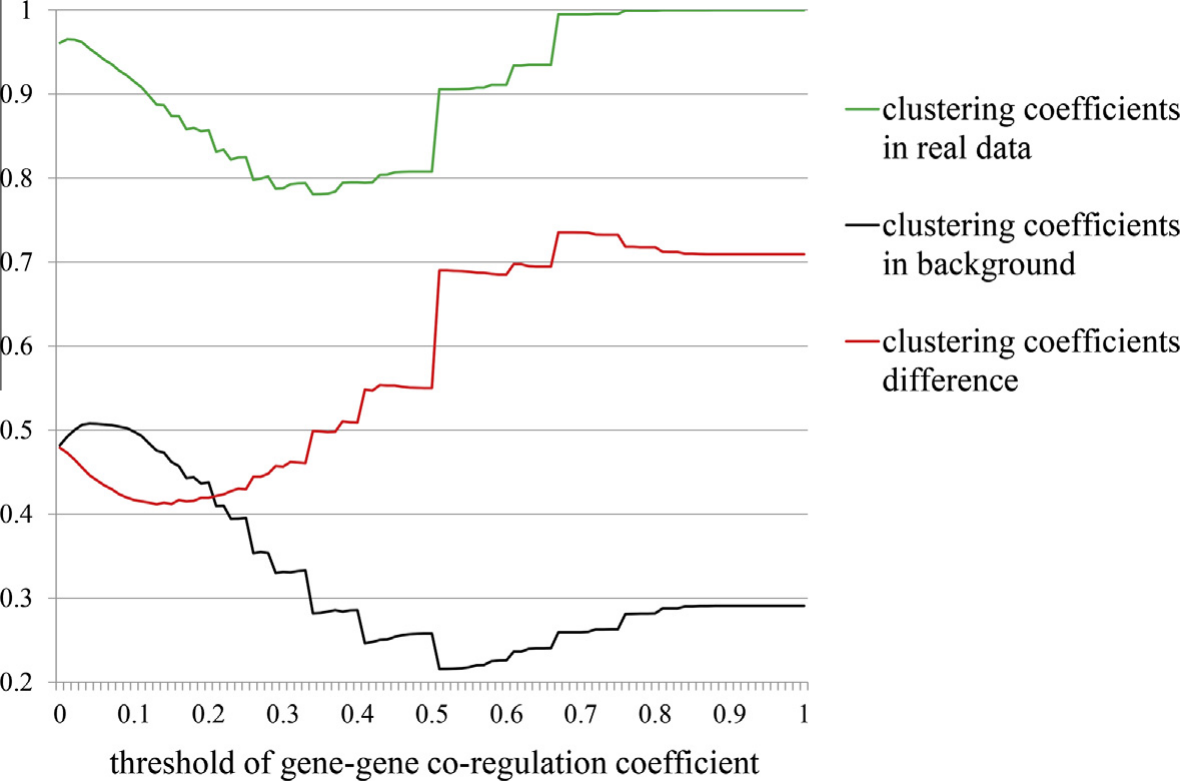
The trends of the clustering coefficients.

From the brain data, we identified 181,906 co-regulated gene pairs of 2830 genes with nonzero co-regulation coefficients. We set the threshold to 0 to 1, a distance of 0.01. We calculated the trends in the clustering coefficients, and found the first extreme point of clustering coefficient difference (the real clustering coefficient minus the background clustering coefficient, Fig. 2). As a result, we identified 25,937 gene pairs of 1444 genes under a threshold value 0.63.

### 3.2 Comparison between HPRD and brain co-regulation network

The comparison of the HPRD PPI and GGCRN network revealed only 4 common gene-gene pairs between 8 genes (Table 1). This indicates that the gene–gene regulation patterns in the GGCRN were different from the protein–protein interactions in the HPRD PPI network. Therefore, the GGCRN can provide new information about gene pairs.

In the following analysis, we used 3 networks, the HPRD PPI network, the GGCRN and the Union network.

### 3.3 Disease gene mining

In the RWR method, to obtain a reasonable restart probability, *r*, we performed a numerical experiment. First, we set *r* to 0, 0.1, 0.2, 0.3, 0.4, 0.5, 0.6, 0.7, 0.8 and 0.9. When *r* was 0, the RWR could not reach steady state, so we added a stop condition of walking no more than 100,000 times. To mine for disease risk genes, we selected the genes with a steady-state probability greater than the initial known disease genes and considered them candidate genes. The results of this analysis are described in Table 2. We found that when *r* was 0, we obtained too many candidate genes and that when *r* was more than 0.1, we obtained too few candidate genes. Then, we set *r* to 0.01, 0.015, 0.02, 0.025, 0.03, 0.035, 0.04, 0.045 and 0.05. We did not know exactly how many disease genes existed; thus we defined a standard for mining risk genes of mining less than 2 times of the known initial genes. For AD, we examined 14 initial genes in the HPRD PPI and Union networks and 4 initial genes in the co-regulation network. Using the co-regulation network, we obtained 41 candidate genes with *r* set at 0.015 and 2 candidate genes with r set at 0.02. As there were only 4 initial genes, this was not a good result, and this is because the co-regulation network is small, only containing 1444 genes. Therefore, it is unsuitable for candidate gene mining using only the GGCRN. Then, we compared the results using the HPRD PPI and Union network. With *r* set at 0.01, the numbers of candidate genes obtained were still insufficient. While, with an *r* value larger than 0.015, we obtained more candidate genes using the HPRD PPI network than the Union network with the same restart probability. In other words, to obtain the same number of candidate genes using these two networks, a higher restart probability is needed for the HPRD PPI network than for the Union network. This indicates that more convergent results can be obtained using the Union network than the HPRD PPI network alone. Therefore, the GGCRN constructed by eQTL data is a useful resource for mining disease genes.

In the DN method, genes with more than 1 linkage with known disease genes were considered candidate disease genes. In the co-regulation network, there were only 4 initial known AD genes, and no gene had more than 1 linkage with known disease genes. In the HPRD PPI and Union networks, there were 14 initial known AD genes, and there were 38 and 39 genes with more than 1 linkage with known disease genes, respectively. These results show that the co-regulation network is not suitable for gene mining by the DN method, because it is too small. Furthermore, the results of gene mining with DN method using the combination of the co-regulation network and the HPRD PPI network were almost the same as those for the HPRD PPI network alone. The detailed results of this analysis are shown in Supplemental Table 2.

For gene mining with the RWR method using the Union network we obtained 27 candidate genes with *r* set at 0.015, and for gene mining with the DN method using the Union network we obtained 39 genes with more than 1 linkage with known disease genes. Only 1 gene was common between these two gene sets. Even when we included all genes that neighbored known AD genes with the DN method, only 11 genes were common between these two gene sets. These results show that the RWR method tends to identify more indirect neighborhood genes, and this is consistent with the gene function pattern of biological networks: genes that interact indirectly may perform the same functions as those that interact directly. Additionally, it was previously reported that the RWR method has the best performance in terms of precision and recall compared to other neighborhood methods [20,21].

### 3.4 Gene annotation

To test the usefulness of the candidate genes mined by RWR, we compared them to those published in the literature. We first verified the 4 most likely AD risk genes (CP, EP300, TP53, and YWHAG, with a restart probability 0.035 in the HPRD PPI and 0.025 in the Union network). The protein encoded by CP is a metalloprotein that binds most of the copper in plasma and is involved in the peroxidation of Fe(II) transferrin to Fe(III) transferrin. Mutations in this gene cause aceruloplasminemia, which is associated with neurologic abnormalities. Some studies confirmed that ceruloplasmin is increased in patients with AD [37,38]. EP300 encodes the adenovirus E1A-associated cellular p300 transcriptional co-activator protein, which mediates cAMP-gene regulation by binding specifically to phosphorylated CREB protein and has been shown to be related to AD [39,40]. TP53 (P53) encodes a tumor suppressor protein that contains transcriptional activation, DNA binding, and oligomerization domains. TP53 is involved in neurodegenerative syndromesis, and is up-regulated in AD [41–43]. YWHAG has been shown to interact with RAF1 and protein kinase C and is involved in various signal transduction pathways. A gene expression analysis showed that YWHAG is down-regulated in AD [44]. These results indicate that these 4 genes are related to AD with high confidence.

To compare between the results of gene mining using the HPRD PPI and Union networks, we analyzed the 27 genes detected in the HPRD PPI with *r* set at 0.02 and the 27 genes detected in the Union network with *r* set at 0.015. The detailed results are shown in Supplemental Table 3. Twenty-two genes were common between the two networks, and they were in front of the 27 genes detected in the HPRD PPI. We then analyzed the 10 genes that were uncommon between the two networks, and we analyzed the ranks of these genes in the results. The 5 genes that only appeared in the results of the HPRD PPI network ranked from 28 to 37 in the results of the Union network, which was just below the level of significance (rank 1–27). However, the 5 genes that only appeared in the results of the Union network ranked from 66 to 547 in the results of the HPRD PPI network. Thus, the 5 genes that only appeared in the results of the Union network are new discoveries based on this gene–gene co-regulation information (Table 3).

We verified these 5 genes by comparison with those published in the literature. RAF1 is the cellular homolog of the viral raf gene (v-raf). It is one of the physiological activators of the ERK pathway, which plays key roles in several steps of tumorigenesis, including cancer cell proliferation, migration, and invasion [45]. Raf-1 activation effectively mediates Ras-dependent signals in AD [46]. The protein encoded by MCM2 is one of the highly conserved mini-chromosome maintenance proteins (MCM) that are involved in the initiation of eukaryotic genome replication. Phosphorylated MCM2 (pMCM2) is markedly associated with neurofibrillary tangles, neuropil threads, and dystrophic neurites in AD [47]. RPS6KA1 encodes a member of the RSK family of serine/threonine kinases. This kinase contains 2 nonidentical kinase catalytic domains and phosphorylates various substrates, including members of the mitogen-activated kinase (MAPK) signaling pathway. Deviation from the strict control of MAPK signaling pathways has been implicated in the development of many human diseases, including AD[45]. MDM2 encodes a nuclear-localized E3 ubiquitin ligase. The encoded protein can promote tumor formation by targeting tumor suppressor proteins, such as p53, for proteasomal degradation. This gene is transcriptionally regulated by p53 [48], which is involved in neurodegenerative syndromes, and is up regulated in AD [41–43]. MAP3K3 (mitogen-activated protein kinase kinase kinase 3) is a 626-amino acid polypeptide. This protein directly regulates the stress-activated protein kinase (SAPK) and extracellular signal-regulated protein kinase (ERK) pathways. MAP3K3 phosphorylates RCAN1, and RCAN1-1L is overexpressed in neurons in AD patients [49]. Furthermore, two polymorphisms of the RCAN1 gene are associated with AD [50]. These results indicated that these 5 genes are related to AD.

Compared with using the HPRD PPI network alone, more disease genes can be identified by integrating the HPRD PPI and GGCRN networks. The results of this study indicate that eQTL-based gene–gene regulation information is a useful resource for disease gene mining.

## 4 Conclusion

There are certain relationships between genes that are associated with the same SNPs. The eQTL-based GGCRN developed here provides extra gene–gene interaction information than the HPRD PPI network. We integrated the GGCRN and HPRD PPI networks and used the RWR method and DN method to mine for disease gene candidates. The RWR method identified more indirect neighborhood candidate genes than the DN method. Applying the AD data, compared to using the HPRD PPI network, using the integrated network obtained better results. With the same restart probability, the integrated network provided faster convergence and identified some new disease-related genes.

Therefore, disease gene mining using an RWR approach with an integrated network (HPRD PPI and GGCRN) is an effective method. This eQTL-based GGCRN also has some disadvantages such as its coverage, the genes in this co-regulation network are not sufficient.

## Author contribution statement

J.L. and M.G. conceived the study. J.L., L.W. and Q.D. did most of the experiments. R.Z., X.L., C.W., Z.T., P.X. and M.Z. analyzed the data. All authors reviewed the manuscript.

## Figures and Tables

**Table 1:** Comparisons of the HPRD PPI and GGCRN networks.

Networks	Number of genes	Number of gene pairs	Number of AD genes
HPRD PPI	9605	39,023	14
GGCRN	1444	25,937	4
Intersection of HPRD and GGCRN	8	4	0
Union of HPRD and GGCRN	10,209	64,956	14

**Table 2:** Comparisons of the results of disease gene mining in 3 networks.

*r*	HPRD PPI	GGCRN	Union network
0	5805	1440	7448
0.01	124	41	304
0.015	58	41	27
0.02	27	2	9
0.025	25	2	4
0.03	7	2	2
0.035	4	2	1
0.04	1	2	1
0.045	1	2	1
0.05	1	2	1
0.1	1	2	1
0.2	0	2	0
0.3	0	2	0
0.4–0.9	0	0	0

**Table 3:** The 10 candidate genes uncommon between the HPRD PPI and Union networks.

Genes	Rank in the results of HPRD	Rank in the results of Union network
CTNNB1	23	28
RB1	24	36
TRAF2	25	30
AKT1	26	34
PIK3R1	27	37
RAF1	66	16
MCM2	423	20
RPS6KA1	476	24
MDM2	126	26
MAP3K3	547	27

## Appendix A Supplementary data

Supplemental Table 1 14 genes in HPRD PPI, GGCRN and Union network.

Supplemental Table 2 The candidate genes of DN method.

Supplemental Table 3 The top 27 genes detected in the HPRD PPI and Union networks.
