# Supplementary material for: Mining disease genes using integrated protein–protein interaction and gene–gene co-regulation information
Source: FEBS Open Bio. 2015 Mar 27;5:251–6. doi: 10.1016/j.fob.2015.03.011 (PMC4392065; doi:10.1016/j.fob.2015.03.011)
Supplement: Supplemental Table 1 — 14 genes in HPRD PPI, GGCRN and Union network. [file mmc1.doc]

Supplemental Table 1. 14 genes in the HPRD PPI, GGCRN and union networks

| HPRD PPI | GGCRN | Union network |
| --- | --- | --- |
| A2M | A2M | A2M |
| ACE | BLMH | ACE |
| APBB2 | PAXIP1 | APBB2 |
| APOE | PSEN1 | APOE |
| APP |  | APP |
| BLMH |  | BLMH |
| HFE |  | HFE |
| MPO |  | MPO |
| NOS3 |  | NOS3 |
| PAXIP1 |  | PAXIP1 |
| PLAU |  | PLAU |
| PSEN1 |  | PSEN1 |
| PSEN2 |  | PSEN2 |
| SORL1 |  | SORL1 |
