# Supplementary material for: Mining disease genes using integrated protein–protein interaction and gene–gene co-regulation information
Source: FEBS Open Bio. 2015 Mar 27;5:251–6. doi: 10.1016/j.fob.2015.03.011 (PMC4392065; doi:10.1016/j.fob.2015.03.011)
Supplement: Supplemental Table 3 — The top 27 genes detected in the HPRD PPI and Union networks. [file mmc3.doc]

Supplemental Table 3. The top 27 genes detected in the HPRD PPI and union networks

| Rank | HPRD PPI | Union network |
| --- | --- | --- |
| 1 | TP53 | EP300 |
| 2 | YWHAG | TP53 |
| 3 | EP300 | YWHAG |
| 4 | SRC | SRC |
| 5 | GRB2 | GRB2 |
| 6 | CREBBP | CP |
| 7 | SMAD4 | SMAD4 |
| 8 | SMAD3 | CREBBP |
| 9 | ESR1 | SMAD3 |
| 10 | PRKCA | CASP3 |
| 11 | CSNK2A1 | ESR1 |
| 12 | CP | PRKCA |
| 13 | SMAD2 | CSNK2A1 |
| 14 | MAPK1 | SMAD2 |
| 15 | EGFR | MAPK1 |
| 16 | FYN | RAF1 |
| 17 | TGFBR1 | EGFR |
| 18 | UBQLN4 | FYN |
| 19 | AR | TGFBR1 |
| 20 | PRKACA | MCM2 |
| 21 | ATXN1 | UBQLN4 |
| 22 | CASP3 | AR |
| 23 | CTNNB1 | ATXN1 |
| 24 | RB1 | RPS6KA1 |
| 25 | TRAF2 | PRKACA |
| 26 | AKT1 | MDM2 |
| 27 | PIK3R1 | MAP3K3 |
